# Association of adherence to the Australian Dietary Guidelines with cognitive performance and cognitive decline in the Sydney Memory and Ageing Study: a longitudinal analysis

**DOI:** 10.1017/jns.2021.44

**Published:** 2021-10-01

**Authors:** Xi Chen, Zhixin Liu, Perminder S. Sachdev, Nicole A. Kochan, Henry Brodaty, Fiona O'Leary

**Affiliations:** 1 Dementia Centre for Research Collaboration, School of Psychiatry, Faculty of Medicine, University of New South Wales, Sydney, NSW 2052, Australia; 2 Mark Wainwright Analytical Centre, University of New South Wales, Sydney, NSW 2052, Australia; 3 Centre for Healthy Brain Ageing (CHeBA), School of Psychiatry, University of New South Wales, Sydney, Australia; 4 Nutrition and Dietetics Group, School of Life and Environmental Science, Charles Perkins Centre, Faculty of Science, University of Sydney, Sydney, NSW 2006, Australia

**Keywords:** Cognitive health, Diet quality, Dietary Guide Index, Food consumption, Nutrition epidemiology, ADG, Australian Dietary Guidelines, APOE, apolipoprotein E, DASH, Dietary Approaches to Stop Hypertension, DGI-2013, Dietary Guideline Index, DQES v2, Dietary Questionnaire for Epidemiological Studies Version 2, HEI, Healthy Eating Index, MAS, Memory and Ageing Study, MIND, Mediterranean-DASH Intervention for Neurodegenerative Delay, NESB, non-English-speaking background, WHO, World Health Organization

## Abstract

This study investigated associations of adherence to the Australian Dietary Guidelines (ADG) with cognitive performance and cognitive decline over 6 years. We used longitudinal data from the Sydney Memory and Aging Study comprising 1037 community-dwelling non-demented participants aged 70–90 years. Dietary intake was assessed at baseline using the Dietary Questionnaire for Epidemiological Studies Version 2. Adherence to the ADG was scored using the Dietary Guideline Index 2013 (DGI-2013). Cognition was assessed using neuropsychological tests in six cognitive domains and global cognition at baseline and 2, 4 and 6 years later. Linear mixed models analysed the association between adherence to the ADG and cognitive function and cognitive decline over 6 years. Results indicated that overall adherence to the ADG was suboptimal (DGI-2013 mean score 43⋅8 with a standard deviation of 10⋅1; median score 44, range 12–73 with an interquartile range of 7). The percent of participants attaining recommended serves for the five food groups were 30⋅2 % for fruits, 11⋅2 % for vegetables, 54⋅6 % for cereals, 28⋅9 % for meat and alternatives and 2⋅1 % for dairy consumption. Adherence to the ADG was not associated with overall global cognition over 6 years (*β* = 0⋅000; 95 % CI: −0⋅007, 0⋅007; *P* = 0⋅95). Neither were DGI-2013 scores associated with change in global cognitive performance over 6 years (*β* = 0⋅002; 95 % CI: −0⋅002, 0⋅005; *P* = 0⋅41) nor in any individual cognitive domains. In conclusion, adherence to the ADG was not associated with cognitive health over time in this longitudinal analysis of older Australians. Future research is needed to provide evidence to support specific dietary guidelines for neurocognitive health among Australian older adults.

## Introduction

Increasing numbers of people are living with mild cognitive impairment (MCI) or dementia worldwide^(1–3)^ against the background of increased longevity. Diet is recognised as one of several possible modifiable factors to prevent or delay the onset of cognitive decline in older adults^(4–10)^. Positive effects on cognitive health have been reported, in association with higher adherence to healthy dietary patterns such as the Mediterranean, Dietary Approaches to Stop Hypertension (DASH) and Mediterranean-DASH Intervention for Neurodegenerative Delay (MIND) diets as well as prudent healthy diets generated through data-driven methods.

Among these diets, the Mediterranean diet is the most investigated by numerous studies including the US study Washington/Hamilton Heights-Inwood Columbia Aging Project (WHICAP)^(2,11)^, the Reasons for Geographic and Racial Differences in Stroke Study^(12)^, the Rush Memory and Aging Project (MAP)^(13)^, the Chicago Health and Aging Project (CHAP)^(14)^ and the (male) Health Professionals’ Follow-Up Study^(15)^. Review articles have confirmed that adherence to the Mediterranean diet has been associated with improvements in cognitive health, despite the heterogeneity of scoring systems used and differences in the results^(16)^.

The DASH diet, although less studied than the Mediterranean diet^(17)^, was positively associated with better cognitive function among older adults by studies such as the MAP^(13)^, the Cache County Study^(18)^ and the Nurse's Health Study^(19)^. The MIND diet was also investigated by the MAP^(13,20)^ and the Nurse's Health Study^(21)^ and has been suggested as having the strongest association with less cognitive decline and dementia risk, when compared to Mediterranean and DASH diets^(17)^, but more evidence on the MIND diet is required.

The prudent healthy diet had positive associations with cognitive health in older populations, as reported by the PATH through life project^(22)^ and the Swedish National Study on Aging and Care-Kungsholmen (SNAC-K)^(23)^. A recent systematic review concluded that higher adherence to a dietary pattern that is plant-based, rich in poly-/mono-unsaturated fatty acids and low in processed foods is likely to be beneficial to long-term cognitive performance among older adults^(24)^.

Healthy diets recommended by dietary guidelines of national peak bodies and the World Health Organization (WHO) have also attracted research attention. Mixed cognitive outcomes were reported from cohort studies using the WHO's Healthy Diet Indicator (HDI)^(25)^ or American dietary guideline indexes including the modified Alternative Healthy Eating Index (mAHEI), Healthy Eating Index (HEI)-2005, HEI 2010 and HEI-2015^(24)^.

The Australian Dietary Guidelines (ADG), released by the Australian National Health and Medical Research Council, were developed to guide food selection for general well-being and chronic disease prevention, with evidenced-based recommendations for both the public and health professionals^(26)^. Higher adherence to the ADG, measured by the Dietary Guideline Index (DGI-2013), has been associated with lower risk of hypertension, obesity and type 2 diabetes^(27–29)^, health behaviours and body mass index^(29)^. However, there is little research as to whether greater adherence to the ADG is related to cognitive performance among older Australians^(24,30,31)^. No significant associations have been reported between the DGI-2013 and cognition^(31)^ or brain MRI findings^(30)^ from cross-sectional studies; there has been no longitudinal analysis.

Our present study has two aims: first, to examine the associations of adherence to the ADG with cognitive performance and cognitive decline in an Australian older cohort over 6 years; and secondly, to explore diet quality and consumption of food groups recommended by the ADG among older adults and investigate effects of food components on overall cognition and cognitive decline.

## Methods

### Participants

Participants were from the community-based Sydney Memory and Ageing Study (MAS)^(32)^ , which comprised 1037 individuals aged 70–90 years without dementia recruited between 2005 and 2007 through the electoral roll following a random approach from two local government areas of Sydney, New South Wales, Australia. Exclusion criteria for study entry were insufficient English to complete assessments; Mini-Mental State Examination (MMSE) score of <24 after adjustment for age, education and non-English-speaking background (NESB); psychotic symptoms or a diagnosis of schizophrenia or bipolar disorder, multiple sclerosis, motor neuron disease and developmental disability; progressive malignancy (active cancer or receiving treatment for cancer, other than prostate – non-metastasised and skin cancer); or other medical or psychological conditions that may prevent assessment^(32)^. Participants provided demographic data, completed a detailed interview reporting medical conditions, current medications and years of education. At baseline and after 2, 4 and 6 years, they underwent neuropsychological and medical assessments and donated blood samples for clinical chemistry and genomics. Informants (relatives or close friends of participants) were interviewed by phone or in-person and completed questionnaires by mail, over the 6-year period.

### Ethics statement

The Sydney MAS was approved by the Ethics Committees of the University of New South Wales and the South Eastern Sydney and Illawarra Area Health Service. Written informed consent was obtained from all participants.

### Dietary assessment

Participants’ dietary intake was assessed at baseline via completion of the Dietary Questionnaire for Epidemiological Studies Version 2 (DQES v2). The DQES v2 is a validated food frequency questionnaire (FFQ) for assessing food and nutrient intake of adults in epidemiological studies, including seventy-four food items and six alcoholic beverages as a modification of FFQ developed by the Cancer Council of Victoria for use in an ethnically diverse Australian population^(34–36)^. Using the DQES v2, participants report intake of foods and alcoholic beverages over the past 12 months using ten response options ranging from ‘never’ to ‘3 or more times per day’, with a series of photographs in the FFQ for elucidating usual portion sizes, as well as a section covering intake of six types of alcoholic beverage with ten frequency response options ranging from ‘never’ to ‘every day’. Food intake (g) was adjusted for total energy intake^(37)^. Nutrient intakes were calculated by the Cancer Epidemiology Centre of the Cancer Council in Victoria using an Australian food composition NUTTAB database^(38)^. Participants with implausible energy intake (<500 kcal or >4000 kcal/d)^(33)^ were excluded.

Adherence to the ADG was measured by the DGI-2013, an updated tool composed of eight components to assess the quality of diet and reflect compliance with recommendations of the 2013 ADG^(29)^ (see Supplementary Table S1). Scoring criteria, all based on daily consumption, consisted of the following: diet variety; the five food groups including daily intake of fruits (servings), vegetables/legumes (servings), cereals (frequency of cereal intake and proportion of whole-grain cereal), meat and alternatives (servings lean meat and alternatives such as tofu, eggs, nuts, seeds and legumes), and dairy and alternatives (all dairy products including low-fat dairy); fat intake (poly-/mono-unsaturated fat to total fat ratio) and consumption of energy-dense foods/fluids (total intake of serves of discretionary foods/drinks)^(29)^. Scores ranged between 0 indicating the poorest adherence and 90 indicating the maximum adherence which occurred when participants met age-/sex-specific recommendations. Detrimental factors including discretionary foods (energy-dense foods/drinks) intake received reverse scoring, while diet variety and consumption of foods from the five food groups received positive scoring. The components for water intake or non-alcoholic beverages were not scored, as they were not included in the DQES v2.

The DGI-2013 scoring criteria and serving sizes for the five food groups recommended by the ADG 2013 are listed in Supplementary Tables S1 and S2, respectively. The higher DGI-2013 scores suggest higher overall adherence to the ADG 2013. DGI-2013 total scores were also categorised into quintiles subsequently, representing groups of very low, low, moderate, high and very high adherence to the ADG 2013.

The reporting of this work is compliant with STROBE-nut guidelines (STrengthening the Reporting of OBservational studies in Epidemiology – Nutritional Epidemiology)^(39)^ (Supplementary Material).

### Cognitive assessment

Cognitive assessments were conducted at baseline (wave 1), 2-year (wave 2), 4-year (wave 3) and 6-year (wave 4) follow-up. Trained psychology graduates administered a comprehensive neuropsychological battery according to standard protocols, covering six cognitive domains: attention/processing speed (comprising Digit Symbol-Coding^(40)^ and Trail Making Test (TMT) A^(41)^); language (the Boston Naming Test^(42)^ and Semantic Fluency (Animals)^(41)^); executive function (Controlled Oral Word Association Test^(41)^ and TMT B^(41)^); visuospatial function (the Block Design test^(43)^); verbal memory (using Logical Memory Story A delayed recall^(44)^, Rey Auditory Verbal Learning Test (RAVLT)^(41)^); global memory (verbal tests as aforementioned, and additional visual memory test using Benton Visual Retention Test recognition^(45)^). Raw cognitive scores were converted into *z*-scores using the baseline mean and standard deviation (sd) values for a reference group (selected from 504 MAS participants who were fluent in English by 10 years of age and classified as cognitive normal at baseline). If necessary, the signs of the *z*-scores were reversed so that higher scores reflected better performance. Domain scores were calculated by averaging *z*-scores of the component tests except for the visuospatial domain which is represented by a single test. Global cognition scores were calculated by averaging the domain scores. All domain and global cognition scores were standardised so that the reference group had a mean of 0 and an SD of 1^(32,46)^.

### Other measurements

Participants were interviewed at baseline and each following wave about their medical history including cardiovascular diseases and related risk factors [including hypertension, hypercholesterolaemia, diabetes, atrial fibrillation, smoking, obesity and stroke or transient ischaemic attack (TIA)^(32)^], mental health issues and current medications. Physical examinations were conducted by trained research assistants who measured height and weight, and seated blood pressure. Apolipoprotein E (APOE) genotyping was determined by peripheral blood or saliva deoxyribonucleic acid (Taqman assays, Applied Biosystems Inc., Foster City, CA, USA); genetic susceptibility was defined as ɛ4 carriage^(32)^.

Hypertension was defined as self-reported previous diagnosis, current treatment, or either systolic blood pressure of ≥140 mmHg or diastolic blood pressure of ≥90 mmHg. The history of stroke or TIA was defined by the previous diagnosis. Diabetes was recognised by either having a previous diagnosis or a fasting blood glucose of ≥7⋅0 mmol/l. The history of depression was defined as self-reported previous diagnosis and treatment due to one or more depressive episodes that required attention from a general practitioner, psychologist or psychiatrist. The assessment of physical activities was conducted using self-report questionnaires developed by the MAS^(46)^, and total physical activity scores represent the number of physical activities participated in listed activities including walking, gardening, yoga, gym work, bowls, golf, tennis, swimming, dancing, bicycling, dancing, aerobics and other sports. Body mass index (BMI = weight in kg/height in m^2^) was determined by measured weight and height. MCI^(48)^ and dementia^(49)^ were diagnosed according to international diagnostic criteria at consensus meetings of experienced clinicians comprising psychogeriatricians, neuropsychiatrists and clinical and research neuropsychologists^(46)^. All measurements were conducted at each wave.

### Statistical analysis

Statistical analyses were performed using R version 3.4.3 (R Foundation for Statistical Computing, Vienna, Austria). Linear mixed-effects models were used to examine the associations between adherence to the ADG measured by DGI-2013 scores and food components at baseline, with cognitive decline over time, as a primary outcome. As a secondary exploration, the association between diet and overall cognitive performance across four waves was investigated, in answering the question about the association between diet and average cognition over 6 years. Overall cognitive performance is representative of average cognitive performance over years, which attenuates variability in each single cognitive assessment, and may be helpful when cognition is measured over a relatively short follow-up in community-dwelling and non-demented participants^(51,52)^. Individual domains were analysed in separate models. The interaction of dietary intake by time is included to examine the impact of dietary intake on the trend of cognitive change over time. Model 1 adjusted for age, sex and education (basic model), and model 2 as the final model, additionally adjusted for NESB, physical activity, BMI, hypertension, diabetes, hypercholesterolaemia, history of stroke/TIA, physical activity, smoking, depression, ethnicity and APOE ɛ4 genotype (fully adjusted model). A sensitivity analysis was conducted excluding participants whose baseline global cognition was below the 10th percentile to explore the role of reverse causality. Further exploration was also conducted to investigate each DGI-2013 quintile (analysed as categorical variables) and cognition in the final model, with the lowest quintile as a reference group.

A significance level of 0⋅05 was set for global cognition, and a level of 0⋅01 was set for multiple secondary outcomes including individual cognitive domains to adjust for multiple tests. Mean, sd and percentage values are provided for characteristics of the Sydney MAS cohort as well as stratified by sex. Independent *t*-tests (for continuous variables) and *χ*^2^ tests (for categorical variables) were used to compare group differences in clinical characteristics, dietary intake and cognitive functions, between female and male participants.

Missing values in the dataset were dealt with using multiple imputation by the chained equation (MICE) approach under the assumption of missing at random^(53,54)^ (details of missing data as in Supplementary Table S3). The multilevel imputation (MI) model was used, and 100 imputed data sets were generated. The parameter estimates for the linear mixed-effects model from the imputed datasets were combined to form a single inference following Rubin's rule^(55)^. Variables used in the imputation model included age, sex, education, NESB, BMI, physical activity, CVD risk factors (hypertension, diabetes and hypercholesterolaemia), history of depression, history of stroke, history of TIA, ethnicity and APOE ɛ4. In addition to listed covariates, global cognition and scores in each cognitive domain were included in the MI process. The MI was conducted using R-package MICE^(56–58)^.

The interaction of DGI-2013 scores by sex, age and education was also tested in this present study. Analyses were planned to repeat the following stratification by sex. As we found no evidence of a dietary score and sex interaction, using dietary score × sex as an interaction term (*β* = −0⋅003; 95 % CI: −0⋅010, 0⋅005; *P =* 0⋅498), we did not repeat analyses stratified by sex.

## Results

The cohort of 1037 participants (55⋅2 % female, *n* 572) had an average age of 78⋅8 years, a mean education level of 11⋅6 years (11⋅0 years for women; 12⋅3 years for men) and a mean BMI of 25⋅7 kg/m^2^ (25⋅3 kg/m^2^ for women; 26⋅1 kg/m^2^ for men) at baseline; 15⋅8 % were from NESBs. About 21⋅4 % of participants were carriers of the APOE ɛ4 allele (genotypes ɛ2/4, ɛ3/4 or ɛ4/4) (Table 1).

**Table 1.** Baseline demographic and clinical characteristics of participants from the Sydney Memory and Ageing study (*N* 1037)

Variables	All (*n* 1037) mean/*N* (%)	sd	Females (*n* 572) mean/*N* (%)	sd	Males (*n* 465) mean/*N* (%)	sd
Age (years)	78⋅8	4⋅8	78⋅9	4⋅9	78⋅8	4⋅7
Years of education	11⋅6	3⋅5	11⋅0	3⋅1	12⋅3	3⋅8
BMI (kg/m^2^)	25⋅7	4⋅4	25⋅3	4⋅7	26⋅1	4⋅0
Vitamin D level (nmol/l)	62⋅2	25⋅5	57	24⋅0	68	26⋅0
Total cholesterol (mmol/l)	4⋅7	1⋅0	5	1⋅0	4⋅4	0⋅9
Triacylglycerols (mmol/l)	1⋅1	0⋅5	1⋅1	0⋅5	1⋅1	0⋅6
HDL-chol (mmol/l)	1⋅4	0⋅4	1⋅6	0⋅4	1⋅3	0⋅4
LDL-chol (mmol/l)	2⋅8	0⋅9	2⋅9	0⋅9	2⋅7	0⋅8
CRP (mg/l)	3⋅0	5⋅5	3	5⋅0	3	6⋅0
Vitamin A (umol/l)	3⋅1	0⋅8	3⋅0	0⋅8	3⋅2	0⋅8
Vitamin E (umol/l)	35⋅3	12⋅7	37⋅6	12⋅5	32⋅8	12⋅6
β-carotene (umol/l)	0⋅8	0⋅6	0⋅9	0⋅7	0⋅6	0⋅5
Total energy intake (KJ/d)	6888⋅0	2234⋅2	6086⋅6	1860⋅9	7904⋅0	2256⋅5
History of hypertension, *N* (%)	629 (60⋅7 %)	n/a	359 (62⋅8 %)	n/a	270 (58⋅1 %)	n/a
History of diabetes, *N* (%)	126 (12⋅2 %)	n/a	48 (8⋅4 %)	n/a	78 (16⋅7 %)	n/a
History of hyperlipidaemia, *N* (%)	623 (60⋅1 %)	n/a	344 (60⋅1)	n/a	279 (60⋅1 %)	n/a
History of depression, *N* (%)	163 (15⋅7 %)	n/a	102 (17⋅8 %)	n/a	61 (13⋅1 %)	n/a
History of stroke, *N* (%)	41 (4⋅0 %)	n/a	14 (2⋅4 %)	n/a	27 (5⋅8 %)	n/a
History of TIA, *N* (%)	69 (6⋅7 %)	n/a	40 (7⋅0 %)	n/a	29 (6⋅2 %)	n/a
Current smoker, *N* (%)	381 (36⋅7 %)	n/a	172 (30⋅1 %)	n/a	209 (54⋅9 %)	n/a
Sum of physical activity^a^	1⋅6	1⋅1	1⋅5	1⋅1	1⋅7	1⋅1
APOE ɛ4 genotype, *N* (%)	222 (21⋅4 %)	n/a	118 (20⋅6 %)	n/a	104 (22⋅4 %)	n/a

sd, standard deviation; BMI, body mass index (calculated by BMI = kg/m^2^ where kg is weight in kilograms and m^2^ is height in metres squared); HDL-chol, high-density-lipoprotein cholesterol; LDL-chol, low-density-lipoprotein cholesterol; CRP, C-reactive protein; n/a, not applicable; TIA, transient ischaemic attack; APOE, apolipoprotein E.

a Physical activity scores represent the number of physical activities participated in, regardless of the amount or intensity of participation. The maximum score is 10 points.

As expected, all mean cognitive composite domain scores gradually declined during the 6-year follow-up. Overall male participants were more likely to perform better in visuospatial function tests, while females scored better in memory tests at all waves (Supplementary Table S4). Dietary data were missing for 6⋅9 % (*n* 63 incomplete and *n* 9 misreported); global cognition data were missing for 0⋅5 % (*n* 5) at baseline and 38⋅9 % (*n* 403) at wave 4 (Supplementary Table S3).

### DGI-2013 scores and food components

In general, adherence to the ADG was poor, with the mean DGI-2013 score of 43⋅8 out of a maximum of 90 points (Table 2; Supplementary Table S5). Men consumed more fruits, cereals and proteins, and women consumed more low-fat dairy foods and less discretionary foods.

**Table 2.** Dietary Guideline Index 2013 component scores and percentage meeting dietary guidelines: the Sydney Memory and Ageing Study (*N* 1037)

DGI-2013 component scores (score range)	All (*n* 1037)			Females (*n* 572)			Males (*n* 465)		
	Mean	sd	% meeting requirement	Mean	sd	% meeting requirement	Mean	sd	% meeting requirement
DGI-2013 score (0–90)	43⋅8	10⋅1		44⋅3	10⋅2		43⋅2	10⋅0	
Food Variety (0–10)	7⋅9	2⋅1	35⋅9	7⋅9	2⋅2	35⋅8	8⋅0	2⋅0	35⋅9
Fruits (0–10)	6⋅3	3⋅5	30⋅2	6⋅1	3⋅4	25⋅3	6⋅5	3⋅6	36⋅1
Vegetables (0–10)	4⋅6	2⋅9	1⋅2	4⋅5	2⋅9	1⋅2	4⋅6	3⋅0	1⋅1
Cereal foods									
Total quantity (0–5)	3⋅7	1⋅7	54⋅6	3⋅9	1⋅7	60⋅0	3⋅5	1⋅8	48
Whole-grain ratio (0–5)	2⋅0	1⋅4	72⋅4	2⋅2	1⋅5	75	1⋅8	1⋅4	69⋅1
Meat and alternatives (0–10)	6⋅1	3⋅6	28⋅9	5⋅9	3⋅6	25⋅5	6⋅4	3⋅6	33⋅1
Dairy and alternatives									
a. Total dairy/alternatives (0–5)	2⋅1	1⋅5	2⋅1	2⋅0	1⋅4	1⋅4	2⋅1	1⋅5	3
b. Low-fat dairy ratio (0–5)	1⋅7	1⋅6	61⋅9	1⋅8	1⋅5	65⋅7	1⋅6	1⋅6	57⋅1
Limit saturated fat and small allowance of unsaturated fat									
a. Unsaturated fat ratio (0–10)	5⋅5	2⋅9	61⋅2	5⋅4	2⋅9	58⋅8	5⋅6	2⋅8	64⋅1
Discretionary foods (0–20)	3⋅3	5⋅1	37⋅7	4⋅1	5⋅4	45⋅5	2⋅3	4⋅6	28⋅2

DGI, Dietary Guideline Index; sd, standard deviation.

Fruit intake of ≥2 serves and vegetable intake of ≥5 serves were considered as satisfactory. Cereal food intakes of ≥4⋅5 serves for men and ≥3 serves for women were considered as satisfactory. Total protein intakes of ≥2⋅5 serves for men and ≥2 serves for women were satisfactory. Dairy intakes of ≥3⋅5 serves for men and ≥4 serves for women were satisfactory. Whole-grain consumption equal to or more than half of total cereal intake serves and low-fat dairy equal to or more than half of total dairy intake serves were considered as meeting requirements here. Unsaturated fats/oils ratio of ≥0⋅5 considered as meeting requirements. Discretionary foods of ≤3 serves for men and ≤2⋅5 serves for women were considered as satisfactory.

Among the five food groups, more than half of the participants (54⋅6 %, *n* 566 for total; 60⋅0 %, *n* 343 for women; 48⋅0 %, *n* 223 for men) consumed a satisfactory amount of cereal recommended for the 70 years and older age group. However, daily fruit and vegetable intakes were limited when compared to ADG 2013 recommendations, with only 1⋅2 % (*n* 12) of all participants (1⋅2 %, *n* 7 for women; 1⋅1 %, *n* 5 for men) meeting the daily vegetable (including legumes) recommendations and 30⋅2 % participants (*n* 313 for total; 25⋅3 %, *n* 145 for women; 36⋅1 %, *n* 168 for men) meeting the daily fruit recommendations. Most participants (71⋅1 %, *n* 737) failed to satisfy requirements from the meat and alternatives food group, which excludes high fat and sodium sources, with only 25⋅5 % (*n* 146) of women and 33⋅1 % (*n* 154) of men meeting ADG daily recommendations. Low intakes of dairy products were also observed; only 22 participants (2⋅1 %) met daily requirements, including eight women (1⋅4 %) and 14 men (3⋅0 %). Overall, there was limited ADG adherence and low consumption of all the five food groups (Table 2; Supplementary Table S5).

### Association between the DGI-2013 and cognitive function

DGI-2013 scores were neither associated with overall performance in global cognition over 6 years nor cognitive decline over 6 years (Table 3). Nor were any significant associations found with overall performance or decline in any individual cognitive domains over 6 years. In sensitivity analysis excluding participants in the lowest 10 percentile of cognitive performance at baseline, results remained non-significant (Supplementary Table S6). There was no interaction between DGI-2013 scores with age, sex or education (Supplementary Table S7).

**Table 3.** Association of the Dietary Guideline Index 2013 scores with overall cognitive function and the change of cognitive performance over 6 years in the Sydney Memory and Ageing Study (*N* 1037)

Cognition domains	Model 1			Model 2		
	*β*	95 % CI	*P* value	*β*	95 % CI	*P* value
Attention						
Overall cognitive performance	0⋅001	−0⋅008, 0⋅006	0⋅78	0⋅001	−0⋅006, 0⋅007	0⋅86
Cognitive change	0⋅001	−0⋅003, 0⋅004	0⋅74	0⋅001	−0⋅003, 0⋅004	0⋅75
Language						
Overall cognitive performance	0⋅003	−0⋅011, 0⋅005	0⋅43	0⋅000	−0⋅007, 0⋅007	0⋅96
Cognitive change	0⋅003	−0⋅000, 0⋅007	0⋅09	0⋅003	−0⋅000, 0⋅007	0⋅09
Executive						
Overall cognitive performance	−0⋅001	−0⋅008, 0⋅006	0⋅78	−0⋅000	−0⋅007, 0⋅007	0⋅96
Cognitive change	0⋅001	−0⋅003, 0⋅004	0⋅75	0⋅000	−0⋅003, 0⋅004	0⋅80
Visuospatial						
Overall cognitive performance	0⋅002	−0⋅004, 0⋅007	0⋅52	0⋅002	−0⋅003, 0⋅008	0⋅40
Cognitive change	0⋅001	−0⋅004, 0⋅002	0⋅52	−0⋅001	−0⋅004, 0⋅002	0⋅51
Memory						
Overall cognitive performance	0⋅002	−0⋅009, 0⋅004	0⋅46	−0⋅001	−0⋅015, 0⋅003	0⋅68
Cognitive change	0⋅002	−0⋅001, 0⋅005	0⋅24	0⋅002	−0⋅007, 0⋅005	0⋅24
Verbal						
Overall cognitive performance	0⋅002	−0⋅008, 0⋅004	0⋅47	−0⋅001	−0⋅007, 0⋅005	0⋅67
Cognitive change	0⋅002	−0⋅001, 0⋅005	0⋅21	0⋅002	−0⋅001, 0⋅005	0⋅22
Global						
Overall cognitive performance	0⋅003	−0⋅010, 0⋅005	0⋅48	0⋅000	−0⋅007, 0⋅007	0⋅95
Cognitive change	0⋅002	−0⋅002, 0⋅005	0⋅41	0⋅002	−0⋅002, 0⋅005	0⋅41

CI, confidence interval.

Values are *β* (95 % CI), *n* 1037. In overall cognitive performance, *β* coefficients show that a 1 score increase measured by the Dietary Guideline Index 2013 is associated with higher cognitive score (positive *β*) or lower cognitive score (negative *β*); in the slope of cognitive change over 6 years, β coefficients show that a 1 score increase measured by the Dietary Guideline Index 2013 is associated with faster cognitive decline (positive *β*) or slower cognitive decline (negative *β*). Results were adjusted for age, sex and education for model 1, and fully adjusted with age, sex and education, as well as non-English speaking background, physical activity, ethnicity, BMI, hypertension, diabetes, hypercholesterolaemia, history of stroke/transient ischaemic attack, smoking, depression and apolipoprotein E ɛ4 genotype for model 2.

*P < 0⋅05 for global cognition or *P* < 0⋅01 for individual cognitive domains is significant.

Results remained non-significant in further analysis on the association between DGI-2013 quintiles as categorical variables and cognitive performance over time, where the lowest quintile indicates the lowest adherence and the highest quintile represents the highest adherence to the DGI-2013 (Table 4). Trajectories of cognitive decline by DGI-2013 quintiles are demonstrated (Supplementary Figures S1 and S2). Further analysis of the DGI-2013 food groups^(59)^ (Supplementary Tables S8 and S9) showed that individual food components were not associated with overall cognitive performance or cognitive decline over time.

**Table 4.** Association between adherence to the Australian Dietary Guidelines with overall cognitive function and the change of cognitive performance over 6 years by quintiles (quintiles 1–5, corresponding to very low to very high adherence) of Dietary Guideline Index 2013: the Sydney Memory and Ageing Study (*N* 1037)

Cognition Domains	Quintile DGI-2013	Overall cognitive performance in 6 years			Slope of cognitive change over 6 years		
		*β*	95 % CI	*P* value	*β*	95 % CI	*P* value*
Attention	Quintile 1 (0–35)	Reference	Reference	Reference	Reference	Reference	Reference
	Quintile 2 (36–42)	0⋅06	−0⋅13, 0⋅26	0⋅53	0⋅08	−0⋅03, 0⋅19	0⋅15
	Quintile 3 (43–47)	0⋅03	−0⋅18, 0⋅24	0⋅77	0⋅07	−0⋅04, 0⋅18	0⋅24
	Quintile 4 (48–52)	−0⋅01	−0⋅22, 0⋅20	0⋅93	0⋅03	−0⋅09, 0⋅15	0⋅59
	Quintile 5 (53–90)	0⋅05	−0⋅15, 0⋅26	0⋅62	0⋅04	−0⋅07, 0⋅15	0⋅48
Language	Quintile 1 (0–35)	Reference	Reference	Reference	Reference	Reference	Reference
	Quintile 2 (36–42)	0⋅12	−0⋅11, 0⋅36	0⋅31	0⋅02	−0⋅09, 0⋅14	0⋅67
	Quintile 3 (43–47)	0⋅11	−0⋅13, 0⋅35	0⋅35	0⋅06	−0⋅05, 0⋅18	0⋅30
	Quintile 4 (48–52)	0⋅07	−0⋅16, 0⋅30	0⋅56	0⋅10	−0⋅02, 0⋅22	0⋅10
	Quintile 5 (53–90)	−0⋅00	−0⋅24, 0⋅23	0⋅99	0⋅07	−0⋅05, 0⋅18	0⋅28
Executive	Quintile 1 (0–35)	Reference	Reference	Reference	Reference	Reference	Reference
	Quintile 2 (36–42)	0⋅03	−0⋅18, 0⋅24	0⋅80	−0⋅05	−0⋅17, 0⋅08	0⋅46
	Quintile 3 (43–47)	0⋅05	−0⋅18, 0⋅27	0⋅68	0⋅06	−0⋅07, 0⋅19	0⋅40
	Quintile 4 (48–52)	−0⋅02	−0⋅26, 0⋅21	0⋅84	−0⋅05	−0⋅18, 0⋅08	0⋅47
	Quintile 5 (53–90)	0⋅03	−0⋅20, 0⋅25	0⋅82	−0⋅01	−0⋅13, 0⋅12	0⋅93
Visuospatial	Quintile 1 (0–35)	Reference	Reference	Reference	Reference	Reference	Reference
	Quintile 2 (36–42)	0⋅15	−0⋅01, 0⋅31	0⋅07	−0⋅02	−0⋅10, 0⋅07	0⋅70
	Quintile 3 (43–47)	0⋅15	−0⋅02, 0⋅33	0⋅08	−0⋅02	−0⋅10, 0⋅07	0⋅70
	Quintile 4 (48–52)	0⋅02	−0⋅17, 0⋅20	0⋅87	−0⋅02	−0⋅10, 0⋅07	0⋅69
	Quintile 5 (53–90)	0⋅11	−0⋅06, 0⋅28	0⋅20	−0⋅03	−0⋅12, 0⋅06	0⋅54
Memory	Quintile 1 (0–35)	Reference	Reference	Reference	Reference	Reference	Reference
	Quintile 2 (36–42)	0⋅12	−0⋅06, 0⋅31	0⋅19	0⋅04	−0⋅04, 0⋅13	0⋅33
	Quintile 3 (43–47)	0⋅00	−0⋅20, 0⋅21	0⋅99	0⋅08	−0⋅02, 0⋅18	0⋅12
	Quintile 4 (48–52)	0⋅04	−0⋅16, 0⋅24	0⋅70	0⋅05	−0⋅05, 0⋅15	0⋅32
	Quintile 5 (53–90)	−0⋅03	−0⋅22, 0⋅16	0⋅79	0⋅04	−0⋅06, 0⋅14	0⋅40
Verbal	Quintile 1 (0–35)	Reference	Reference	Reference	Reference	Reference	Reference
	Quintile 2 (36–42)	0⋅11	−0⋅08, 0⋅30	0⋅26	0⋅05	−0⋅04, 0⋅14	0⋅25
	Quintile 3 (43–47)	−0⋅00	−0⋅20, 0⋅19	0⋅97	0⋅07	−0⋅01, 0⋅16	0⋅10
	Quintile 4 (48–52)	0⋅04	−0⋅16, 0⋅24	0⋅68	0⋅04	−0⋅05, 0⋅13	0⋅37
	Quintile 5 (53–90)	−0⋅03	−0⋅22, 0⋅15	0⋅72	0⋅04	−0⋅04, 0⋅13	0⋅33
Global	Quintile 1 (0–35)	Reference	Reference	Reference	Reference	Reference	Reference
	Quintile 2 (36–42)	0⋅16	−0⋅05, 0⋅38	0⋅14	0⋅03	−0⋅08, 0⋅14	0⋅65
	Quintile 3 (43–47)	0⋅09	−0⋅12, 0⋅31	0⋅40	0⋅06	−0⋅06, 0⋅17	0⋅32
	Quintile 4 (48–52)	0⋅03	−0⋅20, 0⋅26	0⋅82	0⋅03	−0⋅08, 0⋅14	0⋅62
	Quintile 5 (53–90)	0⋅03	−0⋅19, 0⋅25	0⋅76	0⋅04	−0⋅08, 0⋅16	0⋅51

CI, confidence interval.

Values are *β* (95 % CI), *n* 1037. In overall cognitive performance, *β* coefficients show that individual quintile by the Dietary Guideline Index 2013 is associated with higher cognitive score (positive *β*) or lower cognitive score (negative *β*) with reference to quintile 1; in the slope of cognitive change over 6 years, *β* coefficients show that individual quintile measured by the Dietary Guideline Index 2013 is associated with faster cognitive decline (positive *β*) or slower cognitive decline (negative *β*) with reference to quintile 1.

Results were fully adjusted with age, sex and education, as well as non-English speaking background, ethnicity, BMI, hypertension, diabetes, hypercholesterolaemia, history of stroke/transient ischaemic attack, physical activity, smoking, depression and apolipoprotein E ɛ4 genotype.

* *P*<0⋅05 for global cognition or *P* < 0⋅01 for individual cognitive domains is significant.

## Discussion

To our knowledge, this is the first longitudinal study to investigate associations between adherence to the ADG, cognitive performance and age-related changes in cognitive health in older adults aged greater than 65 years. Overall adherence to the ADG was suboptimal among older adults with limited consumption from each of the five food groups and especially poor for vegetables. We observed no associations between DGI-2013 scores measuring ADG adherence, and overall cognition or cognitive decline over 6 years in the Sydney MAS. No food group scored by the DGI-2013 was related to cognitive health.

Our report of no associations between an index assessing adherence to national dietary guidelines and cognition in ageing populations confirms previous research on adherence to Dietary Guidelines for Americans (measured by HEI)^(14,60)^ and the WHO's recommendations (measured by HDI)^(25)^. It is consistent with an Australian cross-sectional study that reported no significant findings between ADG adherence and cognition or brain MRI measures, in a slightly younger cohort with an average age of 70 years^(30)^. Similarly, a 2019 Australian study reported after the adjustment of major confounders (age, sex, education, urban/rural status and physical activity), and no associations were observed between overall diet quality as assessed by the DGI-2013 and cognitive function measured 4 years later, among participants who were mid and early older aged (mean age: 60 years)^(61)^. By contrast, a lower risk of cognitive decline as measured by MMSE was found among those with the highest dietary quality according to the Dietary Guidelines for Americans as measured by the mAHEI^(62)^ when compared with the poorest compliance. Mixed results may be due to study power, different cognitive outcome measurements using various tools and cohort characteristics including food consumption patterns, food supply and lifestyle. Discrepancies between dietary guidelines and dietary index tools may also play a role in explaining these mixed results, such as recommended serving sizes and whether or not brain-healthy foods^(63)^ are scored separately. For example, nuts and soya protein, as well as the ratio of fish to meat and eggs, were individually scored components of the mAHEI^(62)^, while adherence to the ADG assessed by the DGI-2013 scored legumes and fish with the meat and alternative category and therefore may be less able to discriminate dietary pattern differences.

The ADG are based on evidence for the prevention of a wide range of chronic diseases such as cardiovascular disease, diabetes and obesity and were not specifically designed for cognitive health or the prevention of cognitive decline in older adults. Higher adherence to the ADG, measured by the DGI-2013, has been linked to non-cognitive outcomes including lower risk of obesity and hypertension^(64,65)^, lower waist circumference^(66)^ and fewer depressive symptoms^(67)^ among middle-aged and younger adults; and less frailty^(68)^, better overall health-related quality of life^(69)^ and functional ability^(70)^ in older Australian cohorts. By contrast, we found that higher adherence to the Mediterranean and DASH diets were associated with better cognitive function in visuospatial domains in our cross-sectional analysis in this MAS cohort^(31)^, but no association with the DGI-2013. Differences between the ADG recommendations and the major dietary patterns associated with better cognitive health are presented in Supplementary Table S10. For example, when contrasting the ADG with other dietary patterns beneficial for cognitive health, the ADG encourages similar foods including fruits, vegetables and whole grains. However, foods linked to better brain health such as legumes^(71)^ and nuts^(72)^ are included but without specific recommendations on daily servings or individual scoring in the DGI-2013, while some factors specified as detrimental by brain-healthy diets, such as red meat, are counted positively as an essential protein source in the DGI-2013. Additionally, the mono-/poly-unsaturated fats including olive oil have only a small, recommended allowance (approximately 20 g spread or 14 g oil at maximum) in the ADG^(29)^. Although this recommendation may be compatible with the MIND diet where olive oil was recommended as a primary oil without a specific requirement of daily amount^(20)^, intervention studies, such as the PREDIMED study, support a higher consumption with 1 l/week extra virgin olive oil provided as part of the intervention for participants on a Mediterranean diet^(73)^. Higher dietary intake of mono-/poly-unsaturated fatty acids has been positively linked to better cognitive performance or less cognitive decline^(74–76)^, on the background of the human brain's requirement for fatty acids ^(77,78)^. Further research is needed to investigate practical ways to improve poly-/mono-unsaturated fat intake from natural food sources especially olive oil, in the culturally diverse Australian population^(79)^. The ADG recommends limiting discretionary foods, salt and added sugar which are consistent with diets benefiting cognition. Other foods that may benefit cardiovascular health^(80)^ and cognition during ageing could be emphasised. These include berries^(81,82)^ which are a key component of the MIND diet and have been linked to better cognitive health^(13,63,83,84)^. Diets with low GI ranking and high phenolic content could also be encouraged^(85,86)^. More specific dietary guidelines for cognition may be needed for education and policy around better cognitive health for older adults, with clearer messaging on beneficial and detrimental components to guide food selection and eating behaviour.

Our present study provided insight into the diet quality of older Australians, and health implications for this ageing population, although we acknowledge that patterns may have changed since baseline^(87)^. Despite the majority of participants (Table 2) scoring well on food group intakes for whole-grain cereals (72⋅4 % met requirements), consumption of other food groups was low, especially for vegetables (1⋅2 % met requirements), dairy products (2⋅1 % met requirements) and protein group (28⋅9 % met requirements for lean meat and alternatives). This raises concerns as prolonged low intakes may be associated with deficiencies in vitamins, minerals and beneficial bioactive molecules and negatively affect the quality of life and the ageing process^(88–90)^. It is worth noting that the baseline dietary data in the MAS were collected between 2005 and 2007^(91)^, and that the dietary intake of the older Australian population has changed over time. An analysis based on 1995 and 2011/2012 national surveys showed a significant increase in protein intake (g/kg body weight) and of particular concerns a decline in vegetable consumption and higher alcohol intake^(87)^. Continued monitoring of dietary intake is needed to reveal food quantity, quality and trends of dietary intake among older Australians.

We found no sex differences in revised DGI-2013 scores that reflect overall adherence to the ADG. However, when we compared food group consumption, men consumed a larger number of serves of some foods including fruits, cereals, protein foods; while women appeared to be more health-conscious in food selection, consuming more low-fat dairy products, and performed better in limiting intakes in discretionary foods that were high in salt, sugar and saturated fat. This is consistent with previous studies, showing that older women made healthier food choices than men, possibly due to differences in capacity and interest to obtain nutritional knowledge, and translating this knowledge into actions, self-monitoring, and level of capacity in cooking, and preparing nutritious meals and snacks^(92,93)^.

Our present study has multiple strengths. The MAS is a large population-based older-aged (70–90 years old at baseline) cohort who underwent comprehensive screening and diagnosis for cognitive impairment according to standard clinical criteria, using comprehensive neurocognitive tests in multiple cognition domains. Participants were followed for four waves over 6 years to track cognition and other medical conditions, and the change over time. Multiple imputation to deal with missing data is a better approach to deal with missing observations in both outcome and independent variables with repeated measures^(56)^. Statistical adjustments were made for multiple important confounding factors such as NESB, cardiovascular risk factors, depression, smoking status, physical activity and APOE ɛ4 genotype.

There are limitations. The MAS population is an older Australian cohort and the results may not be generalised to younger groups or other ethnic groups. The dietary assessment tool DQES v2 has limited items (e.g. eighteen vegetables and ten fruits); it does not assess consumption of water and non-alcoholic beverages including sugary drinks, thus accurate data on total fluid intake or total sugar intake (from both food and beverages) were not obtained. Nor does the DQES v2 capture specific brain-healthy items of interest, such as varieties of berries and olive oil intake^(24,51)^. Only baseline dietary data were obtained. Repeated measures across four waves may have been more representative of overall dietary intake during the study period, and follow-up dietary data could have enabled investigation of dietary changes with age. We were unable to obtain a lifelong dietary history, due to the nature of nutrition research and difficulty in tracking dietary intake over a lifetime, especially for older populations. However, a British birth cohort study that investigated dietary patterns over the life course reported no significant changes in eating patterns among older adults when dietary intake was assessed at 36, 43, 53 and 60–64 years old^(94)^, although a Belgian study suggested unstable diet quality over 10 years among middle-aged participants^(95)^. Future studies are needed on long-term diet stability in Australia, where diet and food consumption have been increasingly impacted by multiculturalism, with influences from Asian and European countries^(96,97)^. Other factors that may play a role in the change of an individual's eating patterns are complex, including changes of health status such as a new diagnosis of diabetes, changes in geographic or living environments and significant life events such as death of a spouse or socio-economic changes^(98–100)^.

## Conclusion

This is the first longitudinal analysis to investigate associations between adherence to the ADG and cognitive function over time among older adults. No significance was found in global cognition or any cognitive domains over 6 years’ follow-up. Our results provided insight into the diet quality of this well-characterised Australian ageing population. Future guidelines for better cognitive health and dementia prevention in older adults require further research with large-scale longitudinal studies to investigate life course dietary intake and underlying mechanisms between diet, nutrition and cognition.

## Supplementary material

For supplementary material accompanying this paper visit http://dx.doi.org/10.1017/jns.2021.44. click here to view supplementary material
