# Supplementary material for: Association of adherence to the Australian Dietary Guidelines with cognitive performance and cognitive decline in the Sydney Memory and Ageing Study: a longitudinal analysis
Source: J Nutr Sci. 2021 Oct 1;10:e86. doi: 10.1017/jns.2021.44 (PMC8532065; doi:10.1017/jns.2021.44)
Supplement: Supplementary file 1 [file S2048679021000446sup001.docx]

**Supplementary Table 1. Scoring criteria according to Dietary Guidelines Index (DGI) -2013 for Australian Healthy Eating Guideline adherence using DQES^1-3^**

| **Dietary Guideline** | **Indicator and description** | **Criteria for Maximum Score** | **Minimum Score** | **Maximum score** |
| --- | --- | --- | --- | --- |
| **Enjoy a wide variety of nutritious foods** | Food variety: 2 points awarded for each of the five food groups when at least one serving was consumed per day of that group. | Consumption of at least one serve from each food group | 0 | 10 |
| **Eat plenty of vegetables and fruits** | Servings of vegetables and legumes per day:  (tomato, capsicum, lettuce, cucumber, celery, beetroot, carrot, cabbage, cauliflower, broccoli, spinach, green beans, bean sprouts, pumpkin, onion, garlic, mushrooms, zucchini, avocado, potato, peas, baked beans, tofu, other beans) | >70 year:  M ≥5, F ≥ 5 serves/day | 0 | 10 |
|  | Servings of fruits per day  (including tinned fruit, fruit juice, orange, apple, pear, banana, melon, pineapple, strawberry, apricot, peach, mango) | >=2 serves/day | 0 | 10 |
| **Eat plenty of cereals (including breads, rice, pasta, and noodles), preferably whole-grain** | Consumption of breads and cereals per day (All-Bran, bran flakes, weetbix, cornflakes, porridge, muesli, rice, pasta, and bread including high fiber white bread, white bread, wholemeal bread, rye bread, multi-grain bread) | >70 year:  M ≥4.5, F ≥3  serves/day | 0 | 5 |
|  | Proportion of whole grain/high fiber cereal consumed relative to total cereal. | 100% | 0 | 5 |
| **Include lean meat, fish, poultry, and/or alternatives (including tofu, egg, nuts and seeds, legumes)** | Consumption of meats and alternatives per day  (Beef, veal, chicken, lamb, pork, fish, eggs, baked beans, soybean/tofu, other beans, peas, nuts, peanut butter) | >70 year:  M ≥2.5, F ≥ 2 serves/day | 0 | 10 |
| **Include milks, yoghurts, cheeses, and/or alternatives Reduced-fat varieties should be chosen, where possible** | Consumption of dairy products per day, including cheese (hard cheese, firm cheese, soft cheese, ricotta cheese, cottage cheese, cream cheese, low-fat cheese), yoghurt, milk (Full cream, reduced fat milk, skim milk, and soya milk). | >70 year:  M ≥ 3.5, F ≥ 4 serves/day | 0 | 5 |
|  | Low-fat/reduced-fat dairy to total dairy ratio | 100% | 0 | 5 |
| **Limit saturated fat and moderate total fat intake, small allowance of unsaturated oils, fats or spreads** | Total Poly and Mono unsaturated fat intake to total fat ratio | 100% | 0 | 10 |
| **Prevent weight gain: eat according to your energy needs, limit discretionary foods containing saturated fat,**  **alcohol, added salt, and**  **added sugars** | Consumption of total Energy-dense foods/fluids that are not essential for nutrient requirements and contain too much fat, sugar, and salt. Such as soft drinks, cordials, fruit juice drinks, mayonnaise and dressing, chips, jam, confectionery, chocolate, hamburgers, hot chips, meat pies, pizza, cakes and muffins, pies and pastries, puddings, ice cream, cream, biscuits, and all alcoholic beverages | >70 year:  M = 0, F = 0 serves/day | 0  M ≥ 3, F ≥ 2.5 | 20 |

Notes: M, Male; F, Female.

**References**

1. Thorpe, M.G., et al., A Revised Australian Dietary Guideline Index and Its Association with Key Sociodemographic Factors, Health Behaviors and Body Mass Index in Peri-Retirement Aged Adults. Nutrients, 2016. 8(3): p. 160.
2. McNaughton, S.A., et al., An Index of Diet and Eating Patterns Is a Valid Measure of Diet Quality in an Australian Population. The Journal of Nutrition, 2008. 138(1): p. 86-93.
3. National Health and Medical Research Council (2013) Australian Dietary Guidelines Educator Guide. Canberra: National Health and Medical Research Council.

**Supplementary Table 2. Five food groups and recommended daily servings for older adults (≥70 years old): Australian Dietary Guidelines**

| Essential food groups | Female (serves per day) | Male (serves per day) | Size of a serve |
| --- | --- | --- | --- |
| Vegetables | 5 | 5 | A serve of vegetables is approximately 75g: • ½ cup of cooked green or orange vegetables (for example broccoli, spinach, carrots or pumpkin) • ½ cup cooked, dried or canned beans, peas or lentils (preferably with no added salt) • 1 cup of green leafy or raw salad vegetables • ½ cup of sweetcorn • ½ medium potato other starchy vegetables (for example sweet potato, taro or cassava) • 1 medium tomato |
| Fruits | 2 | 2 | A serve of fruit is about 150g: • 1 medium apple, banana, orange or pear • 2 small apricots, kiwi fruits or plums • 1 cup diced or canned fruit (with no added sugar) • or occasionally as a substitute for other foods in the group • ½ cup (125ml) 100% fruit juice (no added sugar) • 30g dried fruit (for example 4 dried apricot halves or 1½ tablespoons of sultanas) |
| Cereals | 3 | 4.5 | • 1 slice of bread (40g) • ½ medium roll or flat bread (40g) • ½ cup cooked rice, pasta, noodles, barley, buckwheat, semolina, polenta, bulgur or quinoa (75–120g) • ½ cup cooked porridge (about 120g) • 2/3 cup wheat cereal flakes (30g) • ¼ cup muesli (30g) • 3 crispbreads (35g) • 1 crumpet (60g) or a small English muffin/ plain scone (35g). |
| Meat and alternatives | 2 | 2.5 | • 65g cooked lean meat (about 90–100g raw weight of beef, veal, lamb, pork, kangaroo or goat) • 80g cooked poultry (about 100g raw weight of skinless chicken or turkey) • 100g cooked fish fillet (about 115g raw weight) or small can of fish • 2 large eggs (120g) • 1 cup (150g) cooked or canned legumes/beans such as lentils, chick peas or split peas (preferably with no added salt) • 170g tofu • 30g nuts, seeds or peanut or almond butter or tahini or other nut or seed paste (no added salt) |
| Dairy and alternatives | 4 | 3.5 | • 1 cup (250ml) fresh, UHT long-life or reconstituted powdered milk or buttermilk • ½ cup (120ml) evaporated milk • 2 slices, or 4x3x2cm piece (40g) hard cheese • ½ cup (120g) ricotta cheese • ¾ cup (200g tub) yoghurt • 1 cup (250ml) soy beverage |

References: National Health and Medical Research Council (2013) Australian Dietary Guidelines Educator Guide. Canberra: National Health and Medical Research Council.

**Supplementary Table 3. Missing data (Mean±SD): Sydney Memory and Ageing Study (N=1037)**

| Missing data | Global cognition | Attention  Processing speed | Language | Executive | Visuospatial | Memory | Verbal  Memory | Dietary data | Reason of missing data |
| --- | --- | --- | --- | --- | --- | --- | --- | --- | --- |
| Wave 1 | 5  (0.48%) | 16  (1.54%) | 7  (0.68%) | 87  (8.39%) | 3  (0.29%) | 11  (1.06%) | 13  (1.25%) | 72 (6.9%) | N=63 not assessed and N=9 not valid (misreported with implausible energy intake) |
| Wave 2 | 175  (16.9%) | 197  (19.0%) | 165  (15.9%) | 247  (23.8%) | 172  (16.6%) | 184  (17.7%) | 190  (18.3%) | N/A | N=43 deceased; N=6 loss of follow-up; N=76 withdrawn; other (not assessed) |
| Wave 3 | 303  (29.2%) | 318  (30.7%) | 285  (27.5%) | 368  (35.5%) | 302  (29.1%) | 312  (30.1%) | 313  (30.2%) | N/A | N=86 deceased; N=10 loss of follow-up; N=125 withdrawn; other (not assessed) |
| Wave 4 | 403  (38.9%) | 428  (41.3%) | 387  (37.3%) | 457  (44.1%) | 414  (39.9%) | 428  (41.2%) | 428  (41.2%) | N/A | N=136 deceased; N=11 loss of follow-up; N=164 withdrawn; other (not assessed) |

Notes: SD, standard deviation. Wave 1- at baseline; wave 2- 2 year follow up; wave 3- 4 year follow up; wave 4- 6 year follow up.

**Supplementary Table 4. Cognitive performance expressed as z scores over 6 years: the Sydney Memory and Ageing study**

| **Cognition Domains** | **All (n=1032)** |  | **Female (n=569)** |  | **Male (n=463)** |  | **P value^*^** |
| --- | --- | --- | --- | --- | --- | --- | --- |
|  |  |  |  |  |  |  |  |
| **Wave 1** | **Mean** | **SD** | **Mean** | **SD** | **Mean** | **SD** |  |
| Attention/Processing Speed | -0.42 | 1.22 | -0.39 | 1.22 | -0.45 | 1.22 | 0.41 |
| Language | -0.74 | 1.53 | -0.80 | 1.50 | -0.68 | 1.58 | 0.22 |
| Executive | -0.46 | 1.27 | -0.48 | 1.26 | -0.44 | 1.28 | 0.63 |
| Visuo-Spatial | -0.34 | 1.09 | -0.48 | 1.03 | -0.16 | 1.14 | <0.001*** |
| Global Memory | -0.52 | 1.21 | -0.29 | 1.20 | -0.81 | 1.15 | <0.001*** |
| Verbal Memory | -0.47 | 1.18 | -0.20 | 1.17 | -0.80 | 1.11 | <0.001*** |
| Global Cognition | -0.72 | 1.38 | -0.71 | 1.34 | -0.74 | 1.41 | 0.75 |
| **Wave 2** | **All (n=862)** |  | **Female (n=463)** |  | **Male (n=399)** |  |  |
| Attention/Processing Speed | -0.50 | 1.44 | -0.45 | 1.29 | -0.57 | 1.59 | 0.22 |
| Language | -0.88 | 1.55 | -0.92 | 1.52 | -0.84 | 1.58 | 0.49 |
| Executive | -0.64 | 1.54 | -0.58 | 1.48 | -0.71 | 1.61 | 0.27 |
| Visuo-Spatial | -0.35 | 1.16 | -0.49 | 1.15 | -0.17 | 1.15 | <0.001*** |
| Global Memory | -0.60 | 1.28 | -0.31 | 1.26 | -0.96 | 1.22 | <0.001*** |
| Verbal Memory | -0.59 | 1.24 | -0.25 | 1.22 | -0.99 | 1.15 | <0.001*** |
| Global Cognition | -0.86 | 1.53 | -0.79 | 1.45 | -0.94 | 1.62 | 0.17 |
| **Wave 3** | **All (n=734)** |  | **Female (n=394)** |  | **Male (n=340)** |  |  |
| Attention/Processing Speed | -0.68 | 1.46 | -0.60 | 1.49 | -0.78 | 1.43 | 0.09 |
| Language | -0.94 | 1.62 | -0.98 | 1.64 | -0.89 | 1.60 | 0.44 |
| Executive | -0.68 | 1.39 | -0.69 | 1.44 | -0.66 | 1.35 | 0.80 |
| Visuo-Spatial | -0.33 | 1.15 | -0.45 | 1.16 | -0.19 | 1.13 | 0.002** |
| Global Memory | -0.57 | 1.35 | -0.29 | 1.37 | -0.90 | 1.25 | <0.001*** |
| Verbal Memory | -0.55 | 1.30 | -0.22 | 1.30 | -0.94 | 1.19 | <0.001*** |
| Global Cognition | -0.89 | 1.51 | -0.86 | 1.57 | -0.93 | 1.43 | 0.563 |
| **Wave 4** | **All (n=634)** |  | **Female (n=348)** |  | **Male (n=286)** |  |  |
| Attention/Processing Speed | -0.89 | 1.51 | -0.83 | 1.50 | -0.96 | 1.53 | 0.286 |
| Language | -1.03 | 1.68 | -1.08 | 1.72 | -0.97 | 1.64 | 0.278 |
| Executive | -1.04 | 1.98 | -1.03 | 1.84 | -1.05 | 2.15 | 0.897 |
| Visuo-Spatial | -0.54 | 1.22 | -0.65 | 1.19 | -0.41 | 1.24 | 0.013 |
| Global Memory | -0.71 | 1.41 | -0.41 | 1.42 | -1.07 | 1.33 | <0.001*** |
| Verbal Memory | -0.67 | 1.37 | -0.32 | 1.37 | -1.08 | 1.27 | <0.001*** |
| Global Cognition | -1.22 | 1.75 | -1.14 | 1.68 | -1.32 | 1.84 | 0.221 |

**Notes:** statistical significance by gender using independent t test. If necessary, the signs of the z-scores were reversed so that higher scores reflect better performance. Domain scores were calculated by averaging z-scores of the component tests with the exception of the visuo-spatial domain represented by a single test. Global cognition scores were calculated by averaging the domain scores. All domain and global cognition scores were standardised. P < 0.05 for global cognition or P<0.01 for individual cognitive domains, is significant.

**Supplementary Table 5. Consumption of food groups from Australian Dietary Guidelines 2013 and food group differences by sex at baseline: the Sydney Memory and Ageing study (N=1037)**

| **Dietary pattern** | **Mean DGI-2013 Score/**  **serves of Food groups/day**  **(Recommendation of daily serves for older adults** ≥**70 years old)** | **All**  **(N=1037)** | | **Female**  **(N=572)** | | **Male**  **(N=465)** | | **Mean Differences (95% CI)** | **P value^*^** |
| --- | --- | --- | --- | --- | --- | --- | --- | --- | --- |
|  |  | **Mean** | **SD** | **Mean** | **SD** | **Mean** | **SD** |  |  |
| **DGI 2013 (score range 0-90)** | **DGI-2013 score** | 43.8 | 10.1 | 44.3 | 10.2 | 43.2 | 10.0 | -1.11 (-2.39,0.18) | 0.09 |
| **Food group**  **serves/day** | Fruits (≥2 serves/day) | 1.7 | 0.9 | 1.6 | 0.8 | 1.8 | 1.0 | 0.18(0.06, 0.29) | 0.003** |
|  | Vegetables (≥5 serves/day) | 1.9 | 1.0 | 1.9 | 1.0 | 2.0 | 1.1 | -0.26(-0.71, 0.19) | 0.37 |
|  | Grains and cereals  (M ≥ 4.5, F ≥ 3 serves/day) | 4.2 | 2.1 | 3.8 | 2.2 | 4.8 | 1.9 | 0.97(0.71, 1.22) | <0.001*** |
|  | Whole Grains  (mostly whole grain) | 2.6 | 1.6 | 2.4 | 1.5 | 2.8 | 1.7 | 0.35(0.15, 0.55) | 0.001** |
|  | Lean meats or alternatives (M ≥ 2.5, F ≥ 2 serves/day) | 2.0 | 1.6 | 1.7 | 1.2 | 2.4 | 1.9 | 0.73(0.53, 0.93) | <0.001*** |
|  | Dairy products or alternatives  (M ≥ 3.5, F ≥ 4 serves/day) | 1.7 | 0.8 | 1.7 | 0.8 | 1.7 | 0.8 | -0.08(-0.18, 0.03) | 0.14 |
|  | Low fat dairy  (mostly choose low fat) | 0.9 | 0.8 | 1.0 | 0.8 | 0.8 | 0.8 | -0.15(-0.25, -0.04) | 0.008** |
|  | Saturated fats (g)  (trim fat from meat and choose low fat dairy) | 40.6 | 479.6 | 22.4 | 10.4 | 63.3 | 718.1 | 40.95(-19.70, 101.59) | 0.19 |
|  | Unsaturated fats (g)  (M ≤ 2, F ≤ 2 serves/day) | 31.7 | 13.0 | 28.2 | 11.4 | 36.1 | 13.6 | 7.91(6.32, 9.49) | <0.001*** |
|  | Discretionary foods serves/day  (M ≤ 3, F ≤ 2.5 serves/day) | 3.6 | 2.5 | 2.9 | 1.8 | 4.6 | 2.8 | 1.72(1.42, 2.01) | <0.001*** |

Notes: Statistical significance by sex using two sample t test. Abbreviations- M, male; F, female; CI, Confidence Interval. *P < 0.05 is significant; **P < 0.01; ***P < 0.001.

**Supplementary Table 6. A sensitivity analysis excluding participants with baseline cognition in lowest 10^th^ percentile: Association of the Dietary Guideline Index 2013 scores with overall cognitive function and change of cognitive performance over 6 years in the Sydney Memory and Ageing study (N=929)**

| **Cognition Domains** |  | **Model 1** | | | **Model 2** | | | |
| --- | --- | --- | --- | --- | --- | --- | --- | --- |
|  |  | **β** | **95% CI** | ***P value*** | **β** | **95% CI** | | ***P value**** |
| **Attention** | Overall cognitive performance | 0.000 | -0.006, 0.006 | 0.912 | 0.001 | | -0.005, 0.007 | 0.713 |
|  | cognitive change | 0.000 | -0.003, 0.003 | 0.933 | 0.000 | | -0.003, 0.003 | 0.966 |
| **Language** | Overall cognitive performance | -0.002 | -0.010, 0.006 | 0.693 | 0.000 | | -0.007, 0.007 | 0.973 |
|  | cognitive change | 0.002 | -0.002, 0.005 | 0.301 | 0.002 | | -0.001, 0.005 | 0.313 |
| **Executive** | Overall cognitive performance | -0.001 | -0.008, 0.006 | 0.791 | 0.000 | | -0.006, 0.006 | 0.919 |
|  | cognitive change | 0.001 | -0.003, 0.005 | 0.634 | 0.001 | | -0.003, 0.004 | 0.685 |
| **Visuo-Spatial** | Overall cognitive performance | 0.001 | -0.004, 0.007 | 0.607 | 0.002 | | -0.004, 0.007 | 0.538 |
|  | cognitive change | -0.001 | -0.004, 0.002 | 0.472 | -0.001 | | -0.004, 0.002 | 0.447 |
| **Memory** | Overall cognitive performance | -0.001 | -0.007, 0.006 | 0.838 | 0.000 | | -0.006, 0.006 | 0.945 |
|  | cognitive change | 0.002 | -0.001, 0.005 | 0.292 | 0.002 | | -0.001, 0.005 | 0.303 |
| **Verbal** | Overall cognitive performance | -0.001 | -0.007, 0.005 | 0.691 | -0.001 | | -0.007, 0.005 | 0.793 |
|  | cognitive change | 0.002 | -0.001, 0.005 | 0.240 | 0.002 | | -0.001, 0.005 | 0.251 |
| **Global** | Overall cognitive performance | 0.000 | -0.006, 0.006 | 0.924 | 0.001 | | -0.006, 0.008 | 0.812 |
|  | cognitive change | 0.000 | -0.002, 0.002 | 0.547 | 0.000 | | -0.002, 0.002 | 0.593 |

**Notes:** Abbreviations: CI, confidence interval. Values are *β* (95% CI), *n* = 929. In overall cognitive performance, *β* Coefficients show a 1 score increase measured by Dietary Guideline Index-2013 is associated with higher cognitive score (positive *β*) or lower cognitive score (negative *β*) ); in slope of cognitive change over six years, *β* Coefficients show a 1 score increase measured by Dietary Guideline Index-2013 is associated with faster cognitive decline (positive *β*) or slower cognitive decline (negative *β*). Results were adjusted for age, sex, education for model 1; and fully adjusted with age, sex, education, as well as non-English speaking background, physical activity, BMI, hypertension, diabetes, hypercholesterolemia, history of stroke/ transient ischaemic attack (TIA), smoking, depression and APOE ε4 genotype for model 2.

*P < 0.05 for global cognition or P<0.01 for individual cognitive domains, is significant.

**Table 7. Interactions between DGI-2013 scores and cognitive domains by age, sex and education (indicated by p values) in Sydney Memory and Ageing Study (N=1037)**

|  |  |  |  |  |
| --- | --- | --- | --- | --- |
| **Cognition** | **Interaction with DGI-2013** | **β** | **95% CI** | ***P value*** |
|  |  |  |  |  |
| **Attention** | Age | 0.000 | -0.001, 0.000 | 0.576 |
|  | Sex | 0.000 | -0.007, 0.008 | 0.899 |
|  | Education | 0.000 | -0.001, 0.001 | 0.697 |
| **Language** | Age | 0.000 | -0.001, 0.000 | 0.396 |
|  | Sex | 0.000 | -0.008, 0.007 | 0.946 |
|  | Education | -0.001 | -0.002, 0.001 | 0.324 |
| **Executive** | Age | 0.000 | -0.001, 0.001 | 0.861 |
|  | Sex | -0.003 | -0.001, 0.005 | 0.428 |
|  | Education | 0.000 | -0.001, 0.011 | 0.681 |
| **Visuospatial** | Age | 0.000 | -0.000, 0.001 | 0.578 |
|  | Sex | 0.000 | -0.006, 0.005 | 0.898 |
|  | Education | 0.000 | -0.001, 0.001 | 0.724 |
| **Memory** | Age | 0.000 | -0.001, 0.001 | 0.894 |
|  | Sex | -0.001 | -0.007, 0.006 | 0.786 |
|  | Education | 0.000 | -0.001, 0.001 | 0.964 |
| **Verbal Memory** | Age | 0.000 | -0.001, 0.001 | 0.983 |
|  | Sex | -0.001 | -0.007, 0.005 | 0.770 |
|  | Education | 0.000 | -0.001, 0.001 | 0.945 |
| **Global cognition** | Age | 0.000 | -0.001, 0.000 | 0.796 |
|  | Sex | -0.003 | -0.010, 0.005 | 0.498 |
|  | Education | 0.000 | -0.001, 0.001 | 0.776 |

Note: p values<0.05 is significant

**Supplementary Table 8:** **Consumption of food components by Dietary Guideline Index 2013 quintiles: the Sydney Memory and Ageing study (N=1037)**

| **DGI-2013**  **/food group (serves/day)** | **Quintile 1**  **(0-35)** |  | **Quintile 2**  (35-42) |  | **Quintile 3**  (42-47) |  | **Quintile 4**  (47-52) |  | **Quintile 5**  (52-73) |  |
| --- | --- | --- | --- | --- | --- | --- | --- | --- | --- | --- |
|  | **Mean** | **SD** | **Mean** | **SD** | **Mean** | **SD** | **Mean** | **SD** | **Mean** | **SD** |
| DGI score | 29.6 | 5.0 | 39.3 | 2.1 | 45.2 | 1.4 | 49.8 | 1.4 | 57.9 | 4.6 |
| Fruits  (serves/day) | 0.9 | 0.5 | 1.4 | 0.8 | 1.8 | 0.9 | 2.1 | 0.7 | 2.3 | 0.8 |
| Vegetables  (serves/day) | 1.3 | 0.7 | 1.7 | 0.8 | 2.0 | 1.0 | 2.2 | 0.9 | 2.6 | 1.2 |
| Grains and cereals  (serves/day) | 3.4 | 1.5 | 4.1 | 1.8 | 4.5 | 1.9 | 4.6 | 1.7 | 4.7 | 3.0 |
| Whole Grains  (serves/day) | 1.6 | 1.4 | 2.4 | 1.6 | 2.8 | 1.5 | 3.1 | 1.3 | 3.3 | 1.5 |
| Lean meats or alternatives  (serves/day) | 1.3 | 0.7 | 2.0 | 1.5 | 2.1 | 1.6 | 2.3 | 1.3 | 2.4 | 2.1 |
| Dairy products or alternatives  (serves/day) | 1.4 | 0.8 | 1.6 | 0.8 | 1.7 | 0.7 | 1.9 | 0.8 | 2.0 | 0.9 |
| Low fat dairy  (serves/day) | 0.4 | 0.6 | 0.7 | 0.7 | 1.0 | 0.7 | 1.2 | 0.8 | 1.5 | 0.8 |
| Saturated fat (g) | 24.6 | 9.4 | 28.0 | 13.8 | 26.8 | 15.2 | 26.1 | 12.9 | 21.0 | 11.4 |
| Unsaturated fat (g) | 26.3 | 9.2 | 33.8 | 15.3 | 34.9 | 16.1 | 35.9 | 15.3 | 33.2 | 16.4 |
| Discretionary foods  (serves/day) | 4.0 | 1.6 | 4.2 | 2.7 | 4.1 | 3.0 | 3.8 | 2.4 | 2.0 | 1.7 |

Notes: DGI, Dietary Guideline Index; SD, standard deviation.

**Supplementary Table 9. Linear mixed model analyses: Association of consumption of DGI-2013 food components with overall global cognition and change of global cognition over 6 years in the Sydney Memory and Ageing study (N=1037)**

| **Food components**  **(serves/day)** | | **Overall global cognition** | | | **Change in global cognition** | | | |
| --- | --- | --- | --- | --- | --- | --- | --- | --- |
|  |  | **β** | **95% CI** | ***P value*** | **β** | **95% CI** | | ***P value**** |
| **Fruits** | | -0.09 | -0.22,0.03 | 0.14 | 0.02 | | -0.02,0.06 | 0.25 |
| **Vegetables** | | 0.01 | -0.10,0.13 | 0.85 | -0.01 | | -0.05,0.02 | 0.45 |
| **Grains and Cereals** | | 0.01 | -0.06,0.08 | 0.81 | 0.01 | | -0.01,0.03 | 0.54 |
| **Whole grains** | | -0.03 | -0.11,0.05 | 0.40 | 0.01 | | -0.02,0.03 | 0.49 |
| **Lean meat and alternatives** | | 0.01 | -0.06,0.09 | 0.77 | -0.01 | | -0.03,0.02 | 0.75 |
| **Dairy products and alternatives** | | 0.00 | -0.14,0.14 | 0.97 | 0.00 | | -0.04,0.04 | 0.90 |
| **Low fat dairy** | | 0.01 | -0.14,0.16 | 0.94 | 0.00 | | -0.04,0.04 | 0.84 |
| **Saturated fats (g/day)** | | 0.00 | 0.00,0.00 | 0.57 | 0.00 | | 0.00,0.00 | 0.67 |
| **Unsaturated fats (g/day)** | | 0.01 | 0.00,0.02 | 0.27 | 0.00 | | 0.00,0.00 | 0.34 |
| **Discretionary foods** | | -0.01 | -0.07,0.05 | 0.81 | 0.00 | | -0.02,0.02 | 0.96 |

**Notes:** DGI, Dietary Guideline Index; CI, confidence interval. Values are *β* (95% CI), *n* = 1037. In overall cognitive performance, *β* Coefficients show a 1 serve/unit increase in daily consumption of food component is associated with higher cognitive score (positive *β*) or lower cognitive score (negative *β*) ); in slope of cognitive change over six years, *β* Coefficients show a 1 serve/unit increase in daily consumption of food components is associated with faster cognitive decline (positive *β*) or slower cognitive decline (negative *β*). Results were fully adjusted with age, sex, education, as well as non-English speaking background, BMI, hypertension, diabetes, hypercholesterolemia, history of stroke/ transient ischaemic attack (TIA), physical activity, smoking, depression, ethnicity and APOE ε4 genotype.

*P < 0.05 for global cognition or P<0.01 for individual cognitive domains, is significant.

**Supplementary Table 10. Food components in dietary patterns: a comparison between healthy diets recommended by Australian Dietary Guidelines and diets associated with better cognitive health**

| **Diet** | **Fruits** | **Vegetables** | **Cereals** | **Nuts and legumes** | **Fish, poultry** | **Berries** | **Olive oil** | **Red and processed meat** | **Dairy food** | **Alcohol** |
| --- | --- | --- | --- | --- | --- | --- | --- | --- | --- | --- |
| **Australian Dietary Guide** | Encourage  ≥2 serves/d | Encourage  M ≥5, F ≥ 5 serves /d | Encourage wholegrain,  M ≥4.5, F ≥3 serves /d | Not specified, counted as protein food | Not specified, counted as protein food | Not specified, counted as fruits | Small allowance | Counted as protein food  limit processed meat | Encourage  Low fat dairy  M ≥ 3.5, F ≥ 4 serves /d | Limit as discretionary food |
| **Mediterranean diet ^[1]^** | High consumption  ≥3 serves /d | High consumption  ≥4 serves /d | Non-refined cereals (whole grain bread, pasta, rice) ≥32 serves/week | Nuts:  ≥3 serves/ week  Legumes: ≥6 serves/ week | Fish:  ≥6 serves/week  Poultry:  3 or less serves/week | Not specified, counted as fruits | Use of olive oil as main culinary fat | 1 serve / week or less | 1 serve/ d or less | moderate intake of alcohol, preferentially red wine during meal |
| **DASH diet ^[2]^** | Encourage  4-5 serves /d | Encourage  4-5 serves/d | Encourage  2-3 serves/d | Encourage  ≥2 serves/d | Encourage fish as source of unsaturated fats | Not specified, counted as fruits | Encourage as source of mono-unsaturated fats | Limit intake | low fat dairy 2-3 serves/d | Not specified, limit sweetened beverages |
| **MIND diet ^[3]^** | Not specified total fruits,  Focused on berries | High consumption  ≥ 7 serves/ week,  Encourage green leafy vegetables | Wholegrain  ≥3 serves /d | Nuts: ≥5  serves/ week  Beans:  ≥3 serves/ week | Fish: ≥1 serve/ week  Poultry: ≥2 serves/ week | ≥ 2 serves/ week | Use of olive oil as primary source of fat | Limit intake | Limit intakes of butter and margarine, cheese | limited intake of wine (red and white wine, 1 serve or less per day) |

Note: serves /d, serves per day. M, male; F, female. DASH diet, the Dietary Approach to Stop Hypertension diet. MIND diet, the Mediterranean-DASH Intervention for Neurodegenerative Delay diet.

**References:**

1. Panagiotakos, D.B., et al., *Adherence to the Mediterranean food pattern predicts the prevalence of hypertension, hypercholesterolemia, diabetes and obesity, among healthy adults; the accuracy of the MedDietScore.* Prev Med, 2007. **44**(4): p. 335-40.

2. Berendsen, A.A.M., et al., *The Dietary Approaches to Stop Hypertension Diet, Cognitive Function, and Cognitive Decline in American Older Women.* J Am Med Dir Assoc, 2017. **18**(5): p. 427-432.

3. Berendsen, A., et al., *Association of long-term adherence to the mind diet with cognitive function and cognitive decline in American women.* The journal of nutrition, health & aging, 2017. **22**(2): p. 222-229.

4. Chen, X., et al., *Dietary Patterns and Cognitive Health in Older Adults: Findings from the Sydney Memory and Ageing Study.* The journal of nutrition, health & aging, 2021. **25**(2): p. 255-262.


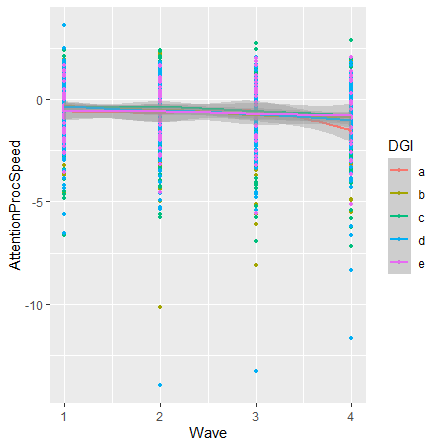

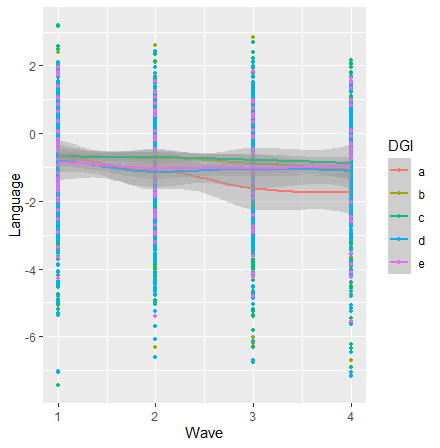

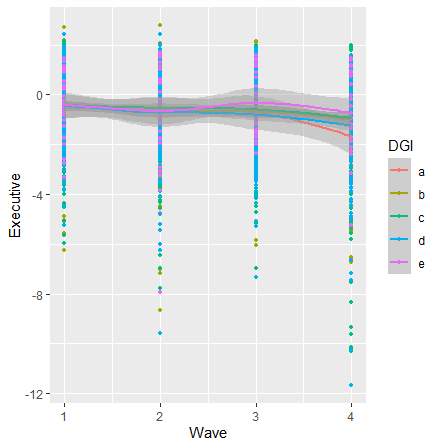

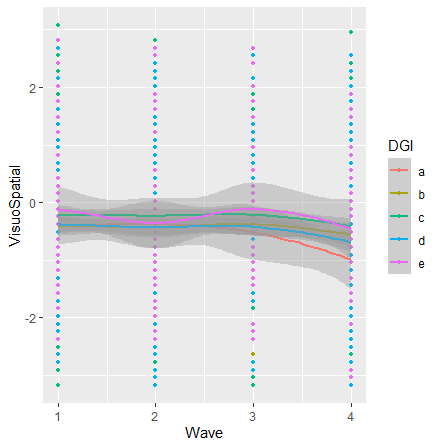

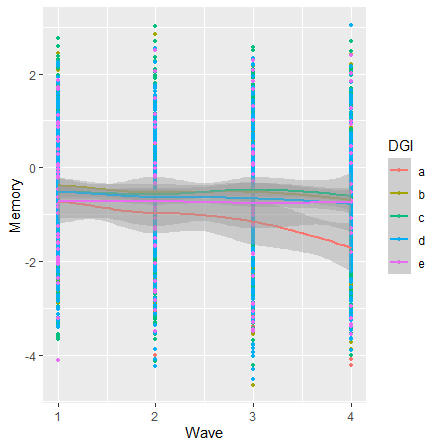

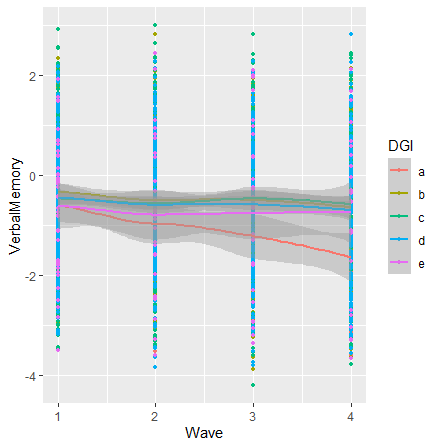


Supplementary Figure 1. Trajectories of cognitive decline in individual domains by DGI-2013 quintiles

Note: a, b, c, d, e are quintiles 1-5 of DGI-2013, where quintile 5 represents highest adherence to Australian Dietary Guidelines


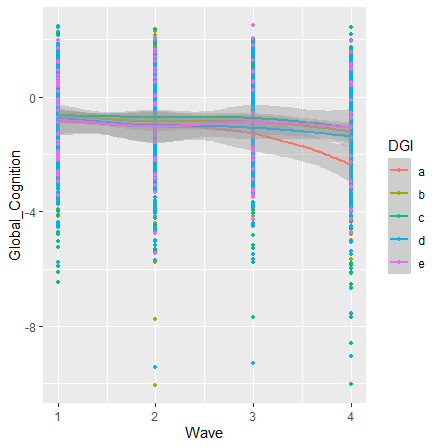


Supplementary Figure 2. Trajectories of cognitive decline in global cognition by DGI-2013 quintiles

Note: a, b, c, d, e are quintiles 1-5 of DGI-2013, where quintile 5 represents highest adherence to Australian Dietary Guidelines

Supplementary Material: STROBE-nut Statement—Checklist of items for nutritional epidemiological cohort studies

|  | Item No | Recommendation | STROBE-nut | Reported on page # |  |
| --- | --- | --- | --- | --- | --- |
| **Title and abstract** | 1 | (*a*) Indicate the study’s design with a commonly used term in the title or the abstract | **nut-1** State the dietary/nutritional assessment method(s) used in the  title, abstract, or keywords. | Title p.1 and abstract p.2 |  |
|  |  | (*b*) Provide in the abstract an informative and balanced summary of what was done and what was found |  | Abstract p.2 |  |
| Introduction | | |  |  |  |
| Background/rationale | 2 | Explain the scientific background and rationale for the investigation being reported |  | Introduction p.3-4 |  |
| Objectives | 3 | State specific objectives, including any prespecified hypotheses |  | Introduction p.3-4 |  |
| Methods | | |  |  |  |
| Study design | 4 | Present key elements of study design early in the paper |  | Methods p.4-5 |  |
| Setting | 5 | Describe the setting, locations, and relevant dates, including periods of recruitment, exposure, follow-up, and data collection | **nut-5** Describe any characteristics of the study settings that might affect the dietary intake or nutritional status of the participants, if applicable. | Method p.4-5 |  |
| Participants | 6 | (*a*) Give the eligibility criteria, and the sources and methods of selection of participants. Describe methods of follow-up | **nut-6** Report particular dietary, physiological or nutritional characteristics that were considered when selecting the target population | Method p.4-5 |  |
|  |  | (*b*) For matched studies, give matching criteria and number of exposed and unexposed |  |  |  |
| Variables | 7 | Clearly define all outcomes, exposures, predictors, potential confounders, and effect modifiers. Give diagnostic criteria, if applicable | **nut-7.1** Clearly define foods, food groups, nutrients, or other food components.  **nut-7.2** When using dietary patterns or indices, describe the methods to obtain them and their nutritional properties. | Method p.4-7 |  |
| Data sources/ measurement | 8* | For each variable of interest, give sources of data and details of methods of assessment (measurement). Describe comparability of assessment methods if there is more than one group | **nut-8.1** Describe the dietary assessment method(s), e.g., portion size estimation, number of days and items recorded, how it was developed and administered, and how quality was assured. Report if and how supplement intake was assessed.  **nut-8.2** Describe and justify food composition data used. Explain the procedure to match food composition with consumption data. Describe the use of conversion factors, if applicable.  **nut-8.3** Describe the nutrient requirements, recommendations, or dietary guidelines and the evaluation approach used to compare intake with the dietary reference values, if applicable.  **nut-8.4** When using nutritional biomarkers, additionally use the STROBE Extension for Molecular  Epidemiology (STROBE-ME). Report the type of biomarkers used  and their usefulness as dietary exposure markers.  **nut-8.5** Describe the assessment of nondietary data (e.g., nutritional status and influencing factors) and timing of the assessment of these variables in relation to dietary assessment.  **nut-8.6** Report on the validity of the dietary or nutritional assessment methods and any internal or external validation used in the study, if applicable. | Method (dietary assessment methods, FFQ used, method to construct dietary pattern scores, Australian Dietary Guide and Dietary Guide Index-2013 used to compare intake with dietary reference values, assessment of non-dietary data, validity of dietary assessment tool) p4-6  Supplementary table 1-2 |  |
| Bias | 9 | Describe any efforts to address potential sources of bias | **nut-9** Report how bias in dietary or  nutritional assessment was addressed, e.g. misreporting, changes in habits as a result of being measured, or data imputation from other sources. | Method p.4-6  Statistical analysis p.7-8 |  |
| Study size | 10 | Explain how the study size was arrived at |  | Method p.4-5 |  |
| Quantitative variables | 11 | Explain how quantitative variables were handled in the analyses. If applicable, describe which groupings were chosen and why | **nut-11** Explain categorization of dietary/nutritional data (e.g., use of N-tiles and handling of nonconsumers) and the choice of reference category, if applicable. | Method p.4-7  Statistical analysis p.7-8 |  |
| Statistical methods | 12 | (*a*) Describe all statistical methods, including those used to control for confounding  (*b*) Describe any methods used to examine subgroups and interactions  (*c*) Explain how missing data were addressed  (*d*) If applicable, explain how loss to follow-up was addressed  (*e*) Describe any sensitivity analyses | **nut-12.1** Describe any statistical method used to combine dietary or nutritional data, if applicable.  **nut-12.2** Describe and justify the method for energy adjustments, intake modeling, and use of weighting factors, if applicable.  **nut-12.3** Report any adjustments for measurement error, i.e,. from a validity or calibration study. | Statistical analysis p.7-8 |  |
| Results | | |  |  |  |
| Participants | 13* | (a) Report numbers of individuals at each stage of study—eg numbers potentially eligible, examined for eligibility, confirmed eligible, included in the study, completing follow-up, and analysed | **nut-13** Report the number of individuals excluded based on missing, incomplete or implausible dietary/nutritional data. | Methods p.4-5  Supplementary material table 3(number of participants, missing data and reasons of missing data) |  |
|  |  | (b) Give reasons for non-participation at each stage |  | Supplementary material table 3 (number of participants, missing data and reasons of missing data) |  |
|  |  | (c) Consider use of a flow diagram |  |  |  |
| Descriptive data | 14* | (a) Give characteristics of study participants (eg demographic, clinical, social) and information on exposures and potential confounders | **nut-14** Give the distribution of participant characteristics across the exposure variables if applicable. Specify if food consumption of total population or consumers only were used to obtain results. | Results p.8-10  Table 1-4  Supplementary Table 4-5  Statistical analysis p.7-8  (demographics and characteristics of study participants and information on exposures and potential confounders as covariates in analysis) |  |
|  |  | (b) Indicate number of participants with missing data for each variable of interest |  | Results p.9  Supplementary material table 3 |  |
|  |  | (c) Summarise follow-up time (eg, average and total amount) |  | Methods p.4-7 |  |
| Outcome data | 15* | Report numbers of outcome events or summary measures over time |  | Methods p.4-7  Results p.8-10  Statistical analysis p.7-8  (outcome events and covariates/models) |  |
| Main results | 16 | (*a*) Give unadjusted estimates and, if applicable, confounder-adjusted estimates and their precision (eg, 95% confidence interval). Make clear which confounders were adjusted for and why they were included  (*b*) Report category boundaries when continuous variables were categorized  (*c*) If relevant, consider translating estimates of relative risk into absolute risk for a meaningful time period | **nut-16** Specify if nutrient intakes are reported with or without inclusion of dietary supplement intake, if applicable. | Statistical analysis p.7-8  Results p.8-10 |  |
| Other analyses | 17 | Report other analyses done—eg analyses of subgroups and interactions, and sensitivity analyses | **nut-17** Report any sensitivity analysis (e.g., exclusion of misreporters or outliers) and data imputation, if applicable. | Statistical analysis p.7-8 |  |
| Discussion | | |  |  |  |
| Key results | 18 | Summarise key results with reference to study objectives |  | Results p.8-10 |  |
| Limitations | 19 | Discuss limitations of the study, taking into account sources of potential bias or imprecision. Discuss both direction and magnitude of any potential bias | **nut-19** Describe the main limitations of the data sources and assessment methods used and implications for the interpretation of the findings. | Discussion p.13 |  |
| Interpretation | 20 | Give a cautious overall interpretation of results considering objectives, limitations, multiplicity of analyses, results from similar studies, and other relevant evidence | **nut-20** Report the nutritional relevance of the findings, given the complexity of diet or nutrition as an exposure. | Discussion p.10-13 |  |
| Generalisability | 21 | Discuss the generalisability (external validity) of the study results |  | Discussion p.10-13 |  |
| Other information | | |  |  |  |
| Funding | 22 | Give the source of funding and the role of the funders for the present study and, if applicable, for the original study on which the present article is based | **nut-22.1** Describe the procedure for consent and study approval from ethics committee(s).  **nut-22.2** Provide data collection tools and data as online material or explain how they can be accessed. | Methods p.5  Funding and author contribution p.14 |  |

By contrast, higher adherence to the Mediterranean and DASH diets, have been associated with better cognitive function in visuospatial domains in a cross-sectional analysis arising from this MAS cohort ^[4]^, concentrate on dietary components that linked to lower risk of cardiovascular disease and to better brain health
